# Late gestational nutrient restriction in primiparous beef females: Performance and metabolic status of lactating dams and pre-weaning calves

**DOI:** 10.1093/jas/skae015

**Published:** 2024-01-19

**Authors:** Colby A Redifer, Lindsey G Wichman, Shelby L Davies-Jenkins, Abigail R Rathert-Williams, Harvey C Freetly, Allison M Meyer

**Affiliations:** Division of Animal Sciences, University of Missouri, Columbia, MO 65211, USA; Division of Animal Sciences, University of Missouri, Columbia, MO 65211, USA; Division of Animal Sciences, University of Missouri, Columbia, MO 65211, USA; Division of Animal Sciences, University of Missouri, Columbia, MO 65211, USA; USDA, ARS, Roman L. Hruska US Meat Animal Research Center, Clay Center, NE 68933, USA; Division of Animal Sciences, University of Missouri, Columbia, MO 65211, USA

**Keywords:** beef calves, beef heifers, developmental programming, lactation, metabolites, pre-weaning growth

## Abstract

Fall-calving primiparous beef females [body weight (**BW**): 451 ± 28 (SD) kg; body condition score (**BCS**): 5.4 ± 0.7] were individually-fed 100% (control; CON; *n* = 13) or 70% (nutrient restricted; NR; *n* = 13) of estimated metabolizable energy and metabolizable protein requirements from day 160 of gestation to calving. Post-calving, all dams were individually-fed tall fescue hay supplemented to meet estimated nutrient requirements for maintenance, growth, and lactation in Calan gates until day 149 of lactation, which limited calves to milk only. From day 150 of lactation until weaning at day 243, dams and calves were group-fed in drylots. Dam BW and metabolic status were determined every 21 d, and BCS and backfat (**BF**) were determined every 42 d of lactation until weaning. Pre-weaning calf BW, size, and metabolic status were determined every 21 d. Data were analyzed with nutritional plane, calving date, and calf sex (when *P *< 0.25) as fixed effects. Circulating metabolites included day and nutritional plane × day as repeated measures. We previously reported that post-calving, NR dams were 64 kg and 2.0 BCS less than CON, but calf BW and size at birth were not affected. During the first 147 d of lactation, NR dams gained more (*P *< 0.01) BW than CON and increased (*P *< 0.01) BCS, while CON decreased (*P *≤ 0.01) BCS and BF. Previously, NR dams had lower (*P* < 0.01) circulating triglycerides on day 1 of lactation, tended to have lower (*P* = 0.08) triglycerides on day 21, and had lower (*P* ≤ 0.04) non-esterified fatty acids (**NEFA**) on days 21 and 243 than CON. Maternal glucose and urea N were not affected (*P *≥ 0.73). At weaning, NR dams weighed 17 kg less (*P *= 0.15), were 0.67 BCS lower (*P *< 0.01), and tended to have less (*P *= 0.06) BF. Calves born to NR dams weighed less (*P *= 0.02) than CON by day 42 of age and were 13% smaller (*P *< 0.01) at weaning. Calf girth measures diverged (*P* ≤ 0.05) by day 21 of age, and skeletal size measures were less (*P* ≤ 0.08) for calves born to NR dams at most timepoints after day 63 of age. Calves born to NR dams tended to have lower (*P *= 0.09) circulating urea N pre-weaning than CON, but glucose, triglycerides, and NEFA were not affected (*P *≥ 0.16). In summary, first-parity beef females that were nutrient restricted during late gestation experienced compensatory growth and gained body condition during lactation but were still thinner at weaning. Nutrient restriction reduced pre-weaning calf growth, likely due to decreased milk production.

## Teaser

First-parity beef females that were nutrient restricted during late gestation and then fed to meet estimated nutrient requirements during lactation experienced compensatory growth but were still in poorer body condition at weaning than controls. Despite a similar birth weight, calves born to nutrient restricted dams diverged in body weight by day 42 of age and weighed less at weaning.

## Introduction

Past research investigating undernutrition during pregnancy has associated placental dysfunction and decreased prenatal nutrient delivery to the fetus with programming adverse postnatal growth, metabolic function, and productivity (Wu et al., 2006; Reynolds et al., 2010). Despite this, a mid- and late gestational nutrient restriction model in primiparous ewes suggested that both prenatal (slower lamb growth rate when raised independently from birth) and postnatal (decreased ewe milk yield) nutrient delivery to offspring can cause reduced postnatal growth (Meyer et al., 2010, 2011). After parturition, maternal physiology often becomes ancillary to research focused on developmental programming effects in the offspring, but the interaction between postpartum nutrition, lactation, and establishment of a subsequent pregnancy is complex (Randel, 1990; McNamara and Shields, 2013). First-parity beef females are expected to continue growing during their first pregnancy and lactation. Even with adequate lactational nutrition, there is competition for nutrients to be partitioned among maintenance, growth, lactation, and initiation of subsequent pregnancy (Bauman and Currie, 1980; Short and Adams, 1988), which likely affects both dam and calf performance and metabolism.

Gestational undernutrition research in beef cattle often involves cow–calf pairs in more extensive settings, allowing calves access to supplementary forage alongside their dams. There is a critical need to conduct more frequent sampling to better understand how quickly nutrient restricted dams recover and the effects on pre-weaning calf growth and metabolism. A thorough study that maintains precise nutritional control of the dams post-calving and limits calves to milk only allows for greater insight into nutrient partitioning in the beef cow–calf system during lactation.

This paper is part of an intensive experiment that investigated the impacts of late gestational nutrient restriction in individually-fed first-parity beef females on prenatal and postnatal nutrient availability and utilization by their calves. We reported that maternal growth was slowed for nutrient restricted heifers during late gestation, and they mobilized maternal stores so that fetal growth was spared (Redifer et al., 2023). Still, calves born to nutrient restricted dams were less vigorous with altered blood chemistry and hematology over the first 48 h of age (Wichman et al., 2023). We hypothesized that dams nutrient restricted during late gestation then fed to meet estimated nutrient requirements during lactation would experience compensatory growth and reach a similar body weight and body condition as control dams by weaning. Furthermore, we hypothesized that late gestational nutrient restriction would alter fetal development and decrease postnatal nutrient delivery through milk, negatively impacting calf pre-weaning performance and metabolism. Our objectives were to investigate the effects of late gestational nutrient restriction in primiparous beef females fed to meet estimated nutrient requirements from calving until weaning on 1) maternal body weight, body condition, and circulating metabolites and 2) calf body weight, size, and metabolic status.

## Materials and Methods

All procedures were approved by the University of Missouri Animal Care and Use Committee (Protocol #9877), and research was conducted at the University of Missouri Beef Research and Teaching Farm (Columbia, MO).

### Animal management and diets during gestation

Animal management and nutritional plane treatments applied during late gestation were described in Redifer et al. (2023). Briefly, 26 single-sired fall-calving Hereford × Simmental–Angus crossbred beef heifers [initial body weight (**BW**) = 451 ± 28 (SD throughout methods) kg, initial body condition score (**BCS**) = 5.4 ± 0.7, calving at 2 yr of age] bred to a single Angus sire were allocated by BW, BCS, fetal sex, and expected calving date to 1 of 2 late gestational nutritional planes from day 160 of gestation to parturition. Control (**CON**; *n* = 13) heifers were individually-fed 100% of estimated metabolizable energy (**ME**) and metabolizable protein (**MP**) requirements for maintenance, pregnancy, and growth, whereas nutrient restricted (**NR**; *n* = 13) heifers were individually-fed 70% of ME and MP requirements. Heifers were housed in 12 partially-covered 3.7 × 15.8 m pens (*n* = 2 or 3 per pen), penned by nutritional plane, and individually-fed via a Calan gate feeding system (Calan Broadbent Feeding System, American Calan, Northwood, NH).

Nutrient requirements were estimated using an expected calf birth weight of 34 kg and projected maternal average daily gain (**ADG**) of 0.36 kg/d. Metabolizable energy for maintenance (**ME**_**m**_) was based on data for heifers in confinement (H. C. Freetly and K. E. Hales, personal communication). The equation used for ME for conceptus was published by Freetly et al. (2005). Equations from NASEM (2016) were utilized for ME for gain (**ME**_**g**_) and MP for maintenance (**MP**_**m**_; Eq. 11-17), conceptus, and gain (**MP**_**g**_). Nutrient requirements were adjusted weekly using the most recent dam BW (recorded every 21 d) and day of gestation.

From days 160 to 265 of gestation, diets were based on ad libitum chopped sorghum sudan hay [1.74 Mcal ME/kg, 6.69% crude protein (**CP**), 72.0% neutral detergent fiber (**NDF**), 52.8% acid detergent fiber (**ADF**); dry matter (**DM**) basis]. Starting on day 266 of gestation, a 3-d transition to ad libitum chopped endophyte-infected tall fescue-based hay (1.90 Mcal ME/kg, 7.22% CP, 65.1% NDF, 43.2% ADF; DM basis) occurred to allow for less supplementation to meet estimated nutrient requirements for the end of gestation and upcoming lactation. Using expected individual hay intakes (estimated from the past week’s hay intakes), heifers were supplemented daily with whole corn, dried distillers’ grains with solubles (**DDGS**), and soyhull pellets to meet their assigned nutritional plane. The supplement for each heifer was formulated and weighed individually. Dams had ad libitum access to water and a trace mineralized salt block (Big 6 Mineral Salt, Compass Minerals America Inc., Overland Park, KS). Beginning on day 274 of gestation, heifers were closely monitored 24 h per day by trained personnel to ensure the time of calving was observed. Peripartum dams and neonatal calves were managed as described in Redifer et al. (2023) and Wichman et al. (2023).

### Animal management and diets during lactation

Following parturition, late gestational nutritional planes were terminated. If calving occurred prior to 0900 h, then the day of calving was considered day 1 of lactation; if calving occurred after 0900 h, then the following day was day 1 of lactation. From days 1 to 149 of lactation, all dams remained in pens with the Calan gate feeding system and were individually-fed 100% of estimated ME and MP requirements for maintenance, lactation, and growth. During this time, calves had access to milk only as they could not access their dams’ diets in the Calan gates. Dams were penned by late gestational nutritional plane, so in the instances where cross-suckling occurred, it was within a nutritional plane. From days 150 to 243 of lactation, dams and calves were managed in drylots, and dams were group-fed to receive 100% of estimated ME and MP requirements for maintenance, lactation, and growth.

During lactation, ME_m_, ME_g_, MP_m_, and MP_g_ were calculated using the same equations and projected maternal ADG as for late gestation. The ME for lactation (**ME**_**l**_) equation from Freetly et al. (2005) was used, which is based on a predicted milk yield equation with an expected peak milk yield of 7.05 kg/d at 10 wk of lactation for primiparous females. The equations for MP for lactation (**MP**_**l**_; Eq. 13-53 and 13-54) were from NASEM (2016) but also used the predicted milk yield equation from Freetly et al. (2005). Requirements were calculated from the following equations; total ME (ME_m_, ME_l_, and ME_g_) and MP (MP_m_, MP_l_, and MP_g_) were then summed.


$$\begin{array}{l} ME_{m}\;(Mcal/d)=0.138{\left(BW\right)}^{0.75} \end{array}$$

$$\begin{array}{l} ME_{l}\;(Mcal/d)=1.0844(predicted\;milk\;yield) \end{array}$$

where predicted milk yield (kg) = 0.000001017t^3^ – 0.000527192t^2^ + 0.059944951t + 5.091;


$$\begin{array}{l} t=day\;of\;lactation \end{array}$$

$$\begin{array}{l} ME_{g}\;(Mcal/d)=4.9 \end{array}$$

$$\begin{array}{l} MP_{m}\;(g/d)=3.8{\left(shrunk\;BW\right)}^{0.75} \end{array}$$

where shrunkBW(kg)=0.96(BW)


$$\begin{array}{l} MP_{l}\;(g/d)=(YProtn/0.65)1,000 \end{array}$$

where YProtn (daily milk protein yield; kg/d) = predicted milk yield(MkProt/100);


$$\begin{array}{lll} \; & predicted\;milk\;yield\;(kg)=\;0.000001017t^{3}-0.000527192t^{2}+\; & 0.059944951t+5.091; \end{array}$$

$$\begin{array}{l} t=day\;of\;lactation; \end{array}$$

$$\begin{array}{l} MkProt\;(milk\;protein\;content;\;\%)=3.4 \end{array}$$

$$\begin{array}{l} MP_{g}\;(g/d)=121.46 \end{array}$$

For the BW used in maintenance requirements, dams that were previously NR had their BW adjusted to a BCS of 5.0. From days 1 to 84 of lactation, NR dam BW was adjusted from a BCS of 3.5 to 5.0 (BW/ 0.894), and from days 85 to 149 of lactation, NR dam BW was adjusted from a BCS of 4.0 to 5.0 (BW/ 0.929) per NASEM (2016; Table 13-3). Estimated MP requirements were converted to CP requirements by the equation CP (g/d) = MP/ 0.64, assuming 80% true protein × 80% digestibility (NASEM, 2016).

#### Individual feeding

From days 1 to 149 of lactation, diets were based on ad libitum chopped endophyte-infected tall fescue-based hay (1.93 Mcal ME/kg, 8.00% CP, 63.6% NDF, 42.0% ADF; DM basis). Individual requirements for ME and CP were adjusted weekly using the most recent dam BW (recorded every 21 d) and day of lactation. Using expected individual hay intakes (estimated from the past week’s hay intakes), dams were supplemented daily with whole corn (3.18 Mcal ME/kg, 8.80% CP; DM basis), DDGS (3.22 Mcal ME/kg, 31.7% CP; DM basis), and soyhull pellets (2.89 Mcal ME/kg, 10.7% CP; DM basis) to meet their estimated nutrient requirements. The supplement for each dam was reformulated weekly to provide between 98 and 102% of each dam’s ME and CP targets, and the supplement for each day was weighed individually.

At the end of gestation, all dams were offered tall fescue-based hay at 1.87% of BW on a DM basis, which allowed for ad libitum feeding. For the first 3- or 4-d feeding period following parturition, hay allocation and supplement formulation used the expectation that hay intake would increase by 0.10% or 0.15% BW on a DM basis over an individual’s last gestational hay intake for NR and CON dams, respectively. We expected that all lactating dams would increase DM intake (**DMI**) post-calving, but that NR dams would experience greater substitution effects because their supplement amounts increased more than CON. Dams that were previously NR received 25, 50, 75, and 100% of the increased amount of supplement on days 1, 2, 3, and 4 of lactation, respectively; CON received 100% of their supplement starting on day 1 of lactation because supplement amount changed minimally. Tall fescue hay was offered at the individual dam’s estimated hay intake + 15% waste. Additionally, to ensure ad libitum intake and reasonable amounts of refusals, the amount of chopped hay offered was adjusted at either daily feeding by 0.45 or 0.90 kg as fed as deemed necessary. The maximum amount offered at 1 feeding was set at 7.72 kg as fed due to space constraints in the Calan gate feeding system. Hay refusals were weighed back twice weekly, and even in instances where the maximum amount of hay was offered, hay refusals were present, indicating ad libitum feeding.

Nutrient analysis of the hay and supplement feedstuffs was conducted throughout the experiment to reformulate the daily supplement to meet ME and CP targets as described for late gestation by Redifer et al. (2023). Representative subsamples of the chopped hay, supplement feedstuffs, and hay refusals were also collected and analyzed after the experiment to calculate nutrient intakes during individual feeding as reported in Redifer et al. (2023). Dry matter, ME, and CP intakes from each ingredient were calculated and summed by day, then averaged by week.

The supplement was fed every morning at approximately 0700 h in a feed pan to prevent wastage and was consumed prior to morning delivery of hay. Supplement refusals were not left by any dam during individual feeding. Hay was delivered in 2 equal portions to each individual dam every morning (0730) and evening (1900). Dams had ad libitum access to water and a trace mineralized salt block (Big 6 Mineral Salt, Compass Minerals America Inc.). Approximately once monthly, pen floors were scraped clean and rebedded with fresh sawdust. Sawdust or other bedding was added as necessary between cleanings.

#### Group feeding

From days 150 to 243 of lactation, dams and calves were managed as a group (both late gestational nutritional planes commingled) and housed in 18 × 61 m drylots (2 pens combined) described by Duncan and Meyer (2019). Dams and calves had access to the feed bunks and round bale feeders in both pens, and a 3-sided shed was accessible for protection from inclement weather. Dams were provided ad libitum endophyte-infected tall fescue-based hay round bales (1.94 Mcal ME/kg, 9.94% CP, 60.8% NDF, 42.0% ADF; DM basis). Requirements for ME and CP for the group were adjusted every 3 wk using the average most recent dam BW (with no adjustment for BCS) and average day of lactation (median day of that 3 wk period). The supplement for the group was formulated using whole corn, DDGS, and soyhull pellets (same feedstuffs used during individual feeding), based on the assumption that hay DMI was similar to the last calculated hay DMI from the Calan gate period. Supplement was fed every morning at approximately 0800 h in fenceline bunks. Supplement refusals were not left during group feeding. Dams had ad libitum access to water and a mineral and vitamin supplement (MLS #12 MINERA-LIX, Midcontinent Livestock Supplements, Inc., Moberly, MO).

#### Pre-weaning calf management

From birth until day 149 of age, calves had access to milk only while their dams were being individually-fed in Calan gates. Once in the large drylots, calves had access to milk and ad libitum hay; bunk space and dominance of the dams left minimal opportunity for calves to consume supplement.

Calves were treated for parasites with oral fenbendazole (SafeGuard, Merck Animal Health, Madison, NJ) on day 109 ± 11 of age. Just before the first group of dams and calves were turned out to drylots, 1 calf from that group was diagnosed with acute hypomagnesemic tetany and treated in the University of Missouri Veterinary Health Center Food Animal Hospital. Serum Mg concentrations indicated that several calves were below the reference interval for Mg (<1.5 mg/dL). All calves in the first group were orally drenched with Mg sulfate suspended in water for 7 to 10 d post-turnout. Younger calves still remaining in the Calan gate facility were drenched twice weekly until turnout.

All male calves were castrated at day 175 ± 3 of age. Calves were weaned and separated from their dams on day 243 ± 3 of age. Calves were administered a vaccination with *Clostridium chauvoei*, *septicum*, *novyi*, *sordellii*, and *perfringens* types C and D bacterin toxoid (Vision 7 with SPUR, Intervet Inc., Omaha, NE) and a modified live vaccination with infectious bovine rhinotracheitis, bovine viral diarrhea types 1 and 2, parainfluenza 3, and bovine respiratory syncytial virus (Pyramid 5 with Metastim, Boehringer Ingelheim Animal Health USA Inc., St. Joseph, MO) on day 224 ± 8 of age and a booster after weaning on day 246 ± 8 of age.

#### Subsequent rebreeding of dams

According to their calving date, dams were split into 2 groups for synchronization of ovulation using the 7-d CO-Synch + controlled internal drug release protocol. Split-time artificial insemination (**AI**) to a single Angus sire was performed on day 104 ± 14 of lactation. The decision not to target a 365-d calving interval was made to decrease the calving season length and to better match the calving season of the mature cowherd at the research farm. If a dam did not conceive and returned to estrus while still housed in the Calan gate facility, then AI to the same Angus sire was performed. A single Angus sire was placed with the dams when they were commingled as a group in drylots to naturally service the remaining non-pregnant dams. Visual estrus detection was routinely performed, and pregnancy determination was conducted approximately 30 d after a female was bred who had not returned to estrus. Nutrient requirements for the subsequent pregnancy were not accounted for at any time prior to weaning because early pregnancy ME and MP requirements are negligible relative to maintenance, growth, and lactation.

### Lactating dam data collection

While being individually-fed, dam BW and jugular blood samples were collected at 21-d intervals occurring on days 1, 21, 42, 63, 84, 105, 126, and 147 of lactation (2-d BW every 42 d and on day 147 of lactation). While being group-fed, dam BW was collected on days 168, 189, 210, and 243 of lactation (2-d BW for weaning at day 243), and jugular blood samples were collected on days 189 and 243 of lactation. Data collection during individual feeding (days 1 to 147) occurred between 1500 and 1630 h and within ± 2 d of the actual day of lactation, corresponding with 4-h milk yield collections (Redifer et al., 2024). Data collection during group feeding occurred between 1200 and 1800 h (days 168 to 210) or 0700 to 1030 h (day 243) and always within ± 6 d of the actual day of lactation. Dam BCS was assessed (1 to 9 scale, 1 = emaciated, 9 = obese; Wagner et al., 1988) by the same 2 trained technicians on days 1, 42, 84, 126, 168, and 243 of lactation, and scores were averaged. Backfat thickness between the 12th and 13th ribs was measured on days 1, 42, 84, 126, and 243 of lactation as described in Redifer et al. (2023). Maternal ADG, BCS change, and backfat thickness change while being individually-fed and while managed as a group were calculated separately.

### Pre-weaning calf data collection

Calves were naturally reared by their dams, with data collected at birth and during the first 48 h of age described in Redifer et al. (2023) and Wichman et al. (2023), respectively. Calf size at birth was determined pre-suckling (0.9 ± 0.3 h of age). Calf BW was determined on days 7 and 14, every 21 d from days 21 to 210, and on day 243 of age. Consecutive 2-d BW were collected on days 147 and 243 of age to represent the end of consuming milk only and weaning, respectively. Calf ADG from days 1 to 147 of age (milk only) and from days 148 to 243 of age was calculated. From days 7 to 210 of age, calf BW measures were collected between 1200 and 1800 h. On day 243 of age, calf BW measures were collected between 0700 and 1030 h. Body weight on days 7, 14, and 21 was collected on the actual day of age, BW from days 42 to 147 was collected within ± 2 d of the actual day of age, and BW from days 168 to 243 was collected within ± 6 d of the actual day of age. Calf birth weight was measured using an electronic hanging scale, calf BW from days 7 to 42 of age was measured with a digital walk-on platform scale, and calf BW from days 63 to 243 of age was measured in the same squeeze chute on weigh bars that was used for their dams.

Calf size measures were collected on all days BW was determined from day 21 of age to weaning and included heart girth, abdominal girth, flank girth, shoulder to rump length, shoulder height, front cannon bone length, and rear cannon bone circumference as described by Redifer et al. (2023). Calf longissimus muscle area was measured on days 7, 42, 84, 126, 168, and 243 of age, as described by Redifer et al. (2023). Beginning on day 126 of age, an Aloka 17 cm 3.5 MHz linear transducer (UST-5044-3.5) and standoff intended for determining longissimus muscle area in cattle was used. From days 7 to 210 of age, jugular blood samples were collected from calves at all BW determinations.

### Circulating metabolite analyses

For dams and calves, blood was collected, plasma and serum were harvested, and colorimetric assays were used to determine plasma glucose, serum urea N, plasma triglycerides, and serum non-esterified fatty acids (**NEFA**) as described by Redifer et al. (2023). For each assay, samples were analyzed in duplicate, and pooled control samples were used. The intraassay and interassay CV for dam lactational metabolites were 3.2 and 4.3% for plasma glucose, 3.0 and 2.0% for serum urea N, 3.0 and 3.3% for plasma triglycerides, and 3.5 and 4.4% for serum NEFA, respectively. The intraassay and interassay CV for pre-weaning calf metabolites were 3.8 and 4.7% for plasma glucose, 3.0 and 4.4% for serum urea N, 3.3 and 2.6% for plasma triglycerides, and 3.5 and 4.1% for serum NEFA, respectively.

### Statistical analyses

One dam (CON) was completely removed from the study due to late gestational abortion. One calf (CON) was euthanized between days 126 and 147 of age due to severe weight loss and unthrifty appearance. After postmortem necropsy revealed the cause to be renal dysplasia, data for the dam was only included through day 126 of lactation (while the calf was suckling) and data for the calf was only included through day 42 of age (when the calf initially began to act lethargic, exhibited a 41.1°C rectal temperature, and medical treatments began). One calf (CON) died due to complications from surgical castration; thus, data for the dam and calf were only included through day 168 of lactation. One calf (NR) was not included on day 147 of age due to hypomagnesemic tetany. This resulted in CON *n* = 10 to 12 and NR *n* = 13 for maternal data and CON *n* = 10 to 12 and NR *n* = 12 or 13 for calf data at each timepoint.

Conception rate to the initial synchronized split-time AI was analyzed using the GLIMMIX procedure while all other measures were analyzed using the MIXED procedure in SAS 9.4 (SAS Institute Inc., Cary, NC) with late gestational nutritional plane as a fixed effect and animal as the experimental unit. Dam and calf circulating metabolites over time also included day of lactation and the nutritional plane × day interaction as fixed effects. These were considered repeated measures using the majority best-fit covariance structure (based on Akaike Information Criterion, Bayesian Information Criterion, and corrected Bayesian Information Criterion) specific for each variable (chosen from unstructured, compound symmetry, heterogeneous compound symmetry, autoregressive, and heterogeneous autoregressive). For all measures except nutrient intakes, Julian date of calving (or Julian date of treatment initiation for any measures collected on day 1 of lactation or age) and calf sex (if *P* ≤ 0.25) were included as covariates (fixed effects). PROC TTEST was used to test if ADG and change in BCS and backfat thickness within late gestational nutritional planes were different than 0. Significance was considered when *P* ≤ 0.05 and tendencies were considered when 0.05 < *P* ≤ 0.10. In the absence of interactions, main effects were reported. Means were separated using least significant difference and the same *P*-value thresholds.

## Results

### Nutrient intake during lactation

**Figure 1.**
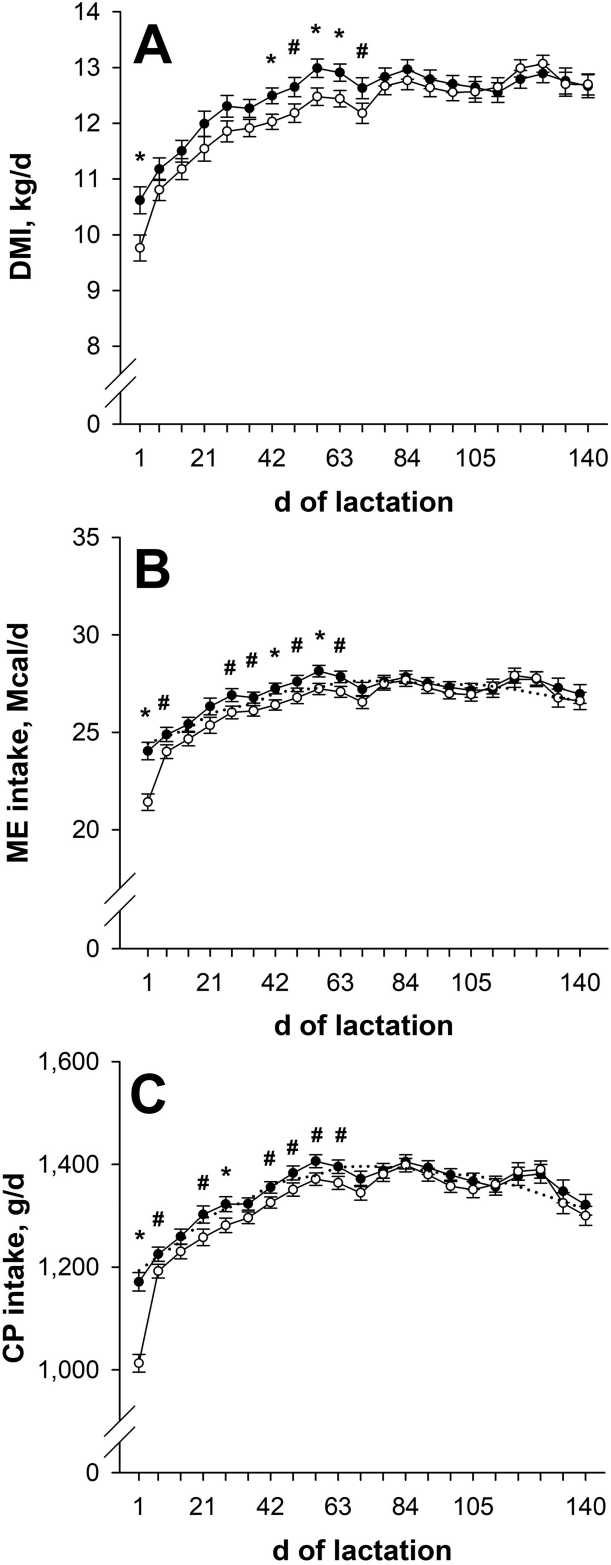
Dry matter intake (DMI; panel A), metabolizable energy intake (ME; panel B), and crude protein intake (CP; panel C) from days 1 to 147 of lactation. Solid circles (●) represent primiparous beef females individually-fed 100% (Control; *n* = 11 or 12) and open circles (○) represent primiparous beef females individually-fed 70% (Nutrient Restricted; *n* = 13) of estimated metabolizable energy and metabolizable protein requirements for maintenance, pregnancy, and growth from day 160 of gestation to parturition. All dams were individually-fed 100% of estimated metabolizable energy and metabolizable protein requirements for maintenance, lactation, and growth from parturition until day 149 of lactation. Least squares means ± SEM are presented. *Nutritional plane means differ (*P* ≤ 0.05). #Nutritional plane means tend to differ (0.05 < *P* ≤ 0.10). Targeted weekly energy and protein intakes are represented by the dotted lines.

Previously NR dams consumed less (*P* ≤ 0.04; Figure 1A) DMI than CON for the weeks beginning on days 1, 42, 56, and 63 and tended to consume less (*P* ≤ 0.10) for the weeks beginning on days 49 and 70 of lactation. Metabolizable energy intake was less (*P* ≤ 0.04; Figure 1B) for the weeks beginning on days 1, 42, and 56 and tended to be less (*P* ≤ 0.10) for the weeks beginning on days 7, 28, 35, 49, and 63 of lactation for NR dams compared with CON. Crude protein intake was less (*P* ≤ 0.05; Figure 1C) for the weeks beginning on days 1 and 28 and tended to be less (*P* ≤ 0.10) for the weeks beginning on days 7, 21, 42, 49, 56, and 63 of lactation for NR dams compared with CON. For CON dams, weekly ME and CP intakes from calving to day 147 of lactation averaged 100.3% (weekly range: 98.2 to 102.9%) and 100.7% (weekly range: 98.4 to 103.2%) of their ME and CP targets, respectively. Weekly ME and CP intakes of NR dams averaged 99.1% (weekly range: 96.1 to 102.9%) of their ME targets and 98.9% (weekly range: 96.3 to 103.6%) of their CP targets from days 7 to 147 of lactation (during the first-week transition to lactation supplement they averaged 87.6% of ME target and 84.9% of CP target). Hay DMI tended to be less (*P* ≤ 0.10; data not shown) for the weeks beginning on days 42, 56, and 63 of lactation for NR dams compared with CON, but hay DMI was not affected (*P* ≥ 0.14) by late gestational nutritional plane at any other time from calving until day 147 of lactation.

### Maternal lactational performance and circulating metabolites

**Figure 2.**
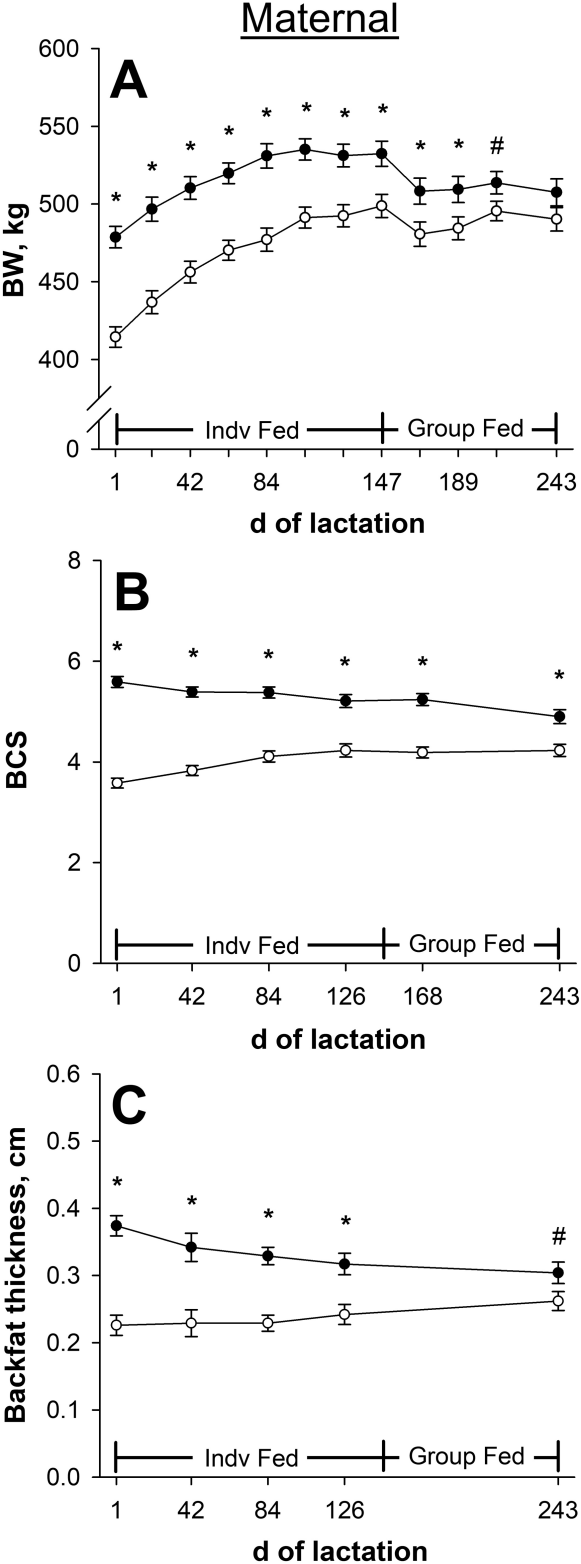
Effects of late gestational nutritional plane on dam body weight (BW; panel A), body condition score (BCS; panel B), and backfat thickness (panel C) from days 1 to 243 of lactation. Solid circles (●) represent primiparous beef females individually-fed 100% (Control; *n* = 10 to 12) and open circles (○) represent primiparous beef females individually-fed 70% (Nutrient Restricted; *n* = 13) of estimated metabolizable energy and metabolizable protein requirements for maintenance, pregnancy, and growth from day 160 of gestation to parturition. All dams were individually-fed 100% of estimated metabolizable energy and metabolizable protein requirements for maintenance, lactation, and growth from parturition until day 149 of lactation and then managed in groups to meet estimated requirements until day 243 of lactation (weaning). Dam BCS was assessed on a 1 to 9 scale (1 = emaciated, 9 = obese). Least squares means ± SEM are presented. *Nutritional plane means differ (*P* ≤ 0.05). #Nutritional plane means tend to differ (0.05 < *P* ≤ 0.10).

Redifer et al. (2023) reported that maternal BW on day 1 of lactation was 13.2% less (*P* < 0.001; 415 vs. 478 ± 7 kg; Figure 2A) for NR dams compared with CON. Maternal BW was less (*P* ≤ 0.006) in previously NR dams than CON for the remainder of individual feeding, but the difference had diminished to 6.2% less (*P* = 0.006; 499 vs. 532 ± 8 kg) on day 147 of lactation. This was due to NR dams having greater (*P* < 0.001; Table 1) maternal ADG from days 1 to 147 of lactation compared with CON when individually-fed to meet estimated ME and MP requirements. Nutrient restricted dams weighed less (*P* ≤ 0.04) on days 168 and 189 and tended to weigh less (*P* = 0.07) on day 210 of lactation compared with CON. Maternal BW was not affected (*P =* 0.15; 490 vs. 508 ± 9 kg) by late gestational nutritional plane on day 243 of lactation. From days 148 to 243 of lactation, dams from both late gestational nutritional planes decreased (*P* ≤ 0.01) BW, but BW loss for NR dams was less (*P* = 0.03; Table 1) than CON during group feeding.

**Table 1.** Effects of late gestational nutritional plane of primiparous beef females on maternal growth and body composition changes during lactation and calf pre-weaning average daily gain

	Late gestational nutritional plane^1^			
Item	Control	Nutrient Restricted	SEM^2^	*P*-value
Maternal average daily gain, kg/d				
Days 1 to 147 of lactation	0.38	0.57	0.03	< 0.001
Days 148 to 243 of lactation	-0.22	-0.09	0.04	0.03
Maternal body condition score change^3^				
Days 1 to 126 of lactation	-0.35	0.68	0.09	< 0.001
Days 127 to 243 of lactation	-0.33	0.002	0.08	0.006
Maternal backfat thickness change, cm				
Days 1 to 126 of lactation	-0.054	0.019	0.017	0.004
Days 127 to 243 of lactation	0.004	0.026	0.018	0.39
Calf average daily gain^4^, kg/d				
Days 1 to 147 of age	0.67	0.55	0.03	0.02
Days 148 to 243 of age	0.92	0.84	0.05	0.08

^1^Primiparous dams were individually-fed either 100% (Control) or 70% (Nutrient Restricted) of estimated metabolizable energy and metabolizable protein requirements for maintenance, pregnancy, and growth from day 160 of gestation to parturition. All dams were individually-fed 100% of estimated metabolizable energy and metabolizable protein requirements for maintenance, lactation, and growth from parturition until day 149 of lactation and then managed in groups to meet estimated requirements until day 243 of lactation (weaning).

^2^Standard error of the mean for Control (*n* = 10 to 12) and Nutrient Restricted (*n* = 12 or 13).

^3^1 to 9 scale (1 = emaciated, 9 = obese).

^4^Calves had access to milk only from days 1 to 149 of age, and then had access to milk and ad libitum tall fescue-based hay from days 150 to 243 of age.

On day 1 of lactation, maternal BCS was 36.0% lower (*P* < 0.001; 3.58 vs. 5.59 ± 0.11; Figure 2B) and maternal backfat thickness was 39.6% less (*P* < 0.001; 0.226 vs. 0.374 ± 0.015 cm; Figure 2C) for NR dams than CON, as reported in Redifer et al. (2023). Dam BCS and backfat thickness were lower (*P* ≤ 0.002) in previously NR dams compared with CON for the remainder of individual feeding, but differences had lessened to 18.8% less (*P* < 0.001; 4.23 vs. 5.21 ± 0.13) BCS and 23.7% less (*P* = 0.002; 0.242 vs. 0.317 ± 0.016) backfat thickness on day 126 of lactation. From days 1 to 126 of lactation, BCS change and backfat thickness change were affected (*P* ≤ 0.004; Table 1) by late gestational nutritional plane. While being individually-fed to meet estimated ME and MP requirements, CON dams decreased (*P* ≤ 0.01) body condition and backfat thickness, while NR dams gained (*P* < 0.001) BCS but had no change (*P* = 0.21) in backfat thickness. Dam BCS was lower (*P* < 0.001) on day 168 of lactation for NR dams compared with CON. On day 243 of lactation, maternal BCS was 13.7% lower (*P* = 0.001; 4.23 vs. 4.90 ± 0.14) and maternal backfat thickness tended to be 13.8% less (*P* = 0.06; 0.262 vs. 0.304 ± 0.016) for NR dams compared with CON. From days 127 to 243 of lactation, BCS change was affected (*P* = 0.006) by late gestational nutritional plane. Control dams decreased (*P* = 0.02) BCS while NR dam BCS did not change (*P* > 0.99) during group feeding. Backfat thickness change from days 127 to 243 of lactation was not affected (*P* = 0.39) by late gestational nutritional plane, and neither CON nor NR dam backfat thickness changed (*P* ≥ 0.14) during group feeding.

**Figure 3.**
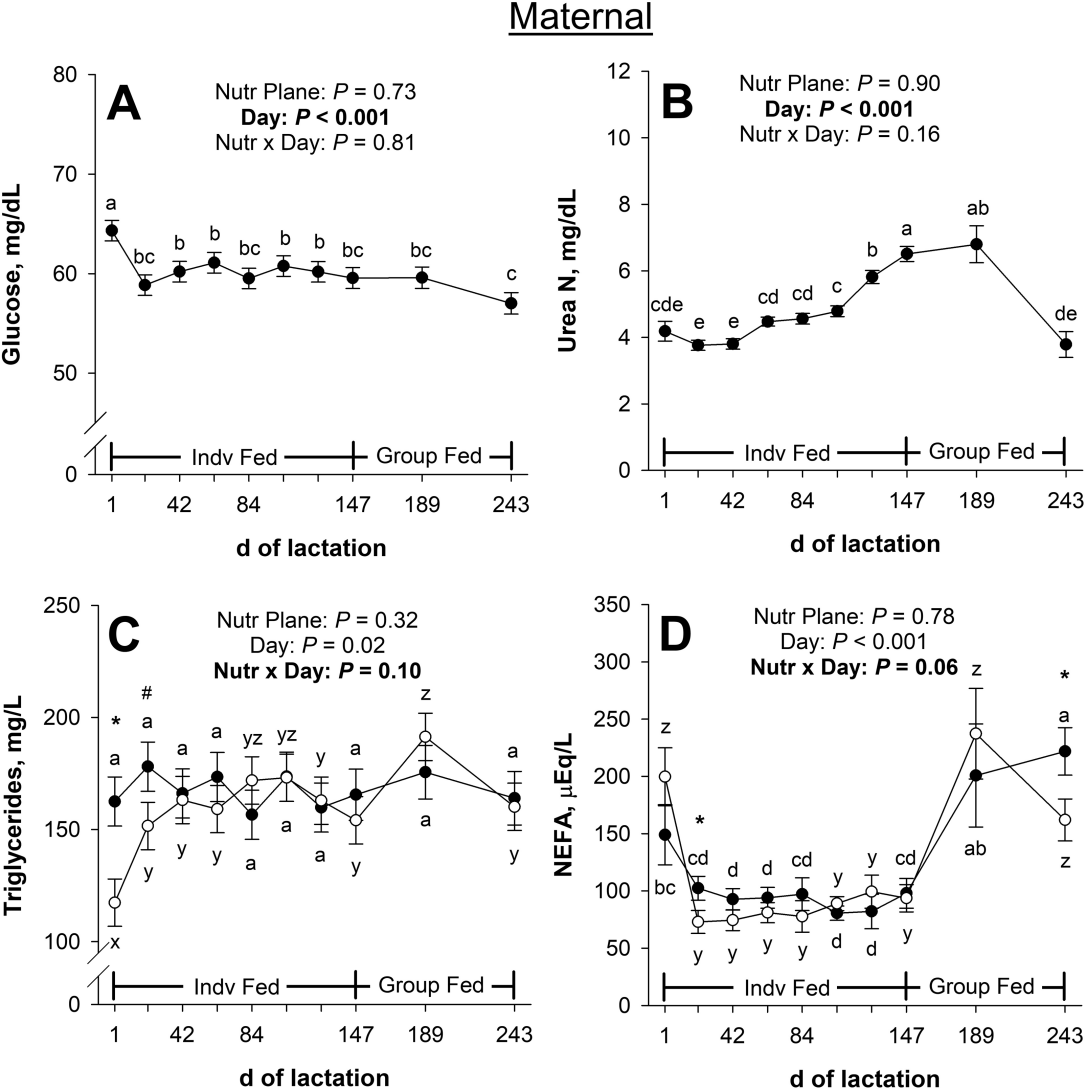
Effects of late gestational nutritional plane on maternal plasma glucose (panel A), serum urea N (panel B), plasma triglycerides (panel C), and serum non-esterified fatty acids (NEFA; panel D) from days 1 to 243 of lactation. Solid circles (●) represent primiparous beef females individually-fed 100% (Control; *n* = 10 to 12) and open circles (○) represent primiparous beef females individually-fed 70% (Nutrient Restricted; *n* = 13) of estimated metabolizable energy and metabolizable protein requirements for maintenance, pregnancy, and growth from day 160 of gestation to parturition. All dams were individually-fed 100% of estimated metabolizable energy and metabolizable protein requirements for maintenance, lactation, and growth from parturition until day 149 of lactation and then managed in groups to meet estimated requirements until day 243 of lactation (weaning). Least squares means ± SEM are presented. *Nutritional plane means within day differ (*P* ≤ 0.05). #Nutritional plane means within day tend to differ (0.05 < *P* ≤ 0.10). ^a,b,c,d,e^Means differ (*P* ≤ 0.05) for main effect of day (or for control across days). ^x,y,z^Means differ (*P* ≤ 0.05) for nutrient restricted across days.

Maternal plasma glucose concentrations during lactation were not affected (*P* ≥ 0.73; Figure 3A) by the late gestational nutritional plane × day interaction or the main effect of nutritional plane. There was an effect (*P* < 0.001) of day of lactation on plasma glucose. Plasma glucose decreased (*P* < 0.001) from days 1 to 21 and tended to decrease (*P* = 0.06) from days 189 to 243 of lactation.

Maternal serum urea N concentrations during lactation were not affected (*P* ≥ 0.16; Figure 3B) by the late gestational nutritional plane × day interaction or the main effect of nutritional plane. Serum urea N was affected (*P* < 0.001) by day of lactation, where urea N increased (*P* ≤ 0.003) from days 42 to 63 and days 105 to 147 but decreased (*P* < 0.001) from days 189 to 243 of lactation.

There tended to be a late gestational nutritional plane × day interaction (*P* = 0.10; Figure 3C) for maternal plasma triglyceride concentrations during lactation. Plasma triglycerides were less (*P* = 0.003) on day 1 and tended to be less (*P* = 0.08) on day 21 of lactation in previously NR dams compared with CON but were not affected (*P* ≥ 0.31) by late gestational nutritional plane for the remainder of lactation. For CON dams, plasma triglycerides did not change (*P* ≥ 0.24) throughout lactation; however, for NR dams, plasma triglycerides increased (*P* ≤ 0.01) from days 1 to 21 and days 147 to 189 but decreased (*P* = 0.03) from days 189 to 243 of lactation.

There tended to be a late gestational nutritional plane × day interaction (*P* = 0.06; Figure 3D) for maternal serum NEFA concentrations during lactation. Serum NEFA were less (*P* ≤ 0.04) in previously NR dams compared with CON on days 21 and 243 of lactation. For CON dams, serum NEFA tended to decrease (*P* = 0.09) from days 1 to 21 but increased (*P* = 0.02) from days 147 to 189 of lactation. For NR dams, serum NEFA decreased (*P* < 0.001) from days 1 to 21, increased (*P* < 0.001) from days 147 to 189, and tended to decrease (*P* = 0.07) from days 189 to 243 of lactation.

Conception rate to the initial synchronized split-time AI [*P* = 0.54; 6 of 12 (CON) vs. 8 of 13 (NR)] and days postpartum at conception (*P* = 0.93; 117 vs. 118 ± 5 d) were not affected by late gestational nutritional plane. Fourteen of the 25 dams conceived to the initial synchronized split-time AI, and the remaining 11 dams conceived to either AI (7 of 11) or natural mating (4 of 11) within 47 d of split-time AI. Twenty-two of the 25 dams had live calves in their second parity, and those that did not were an embryonic loss late in the breeding season (CON), a mid-gestational abortion (CON), and a late gestational unviable premature birth (NR).

### Calf growth and metabolic status through weaning

We reported that calf BW and size measures at birth and calf longissimus muscle area on day 7 of age were not affected (*P* ≥ 0.27) by late gestational nutritional plane (Redifer et al., 2023).

**Figure 4.**
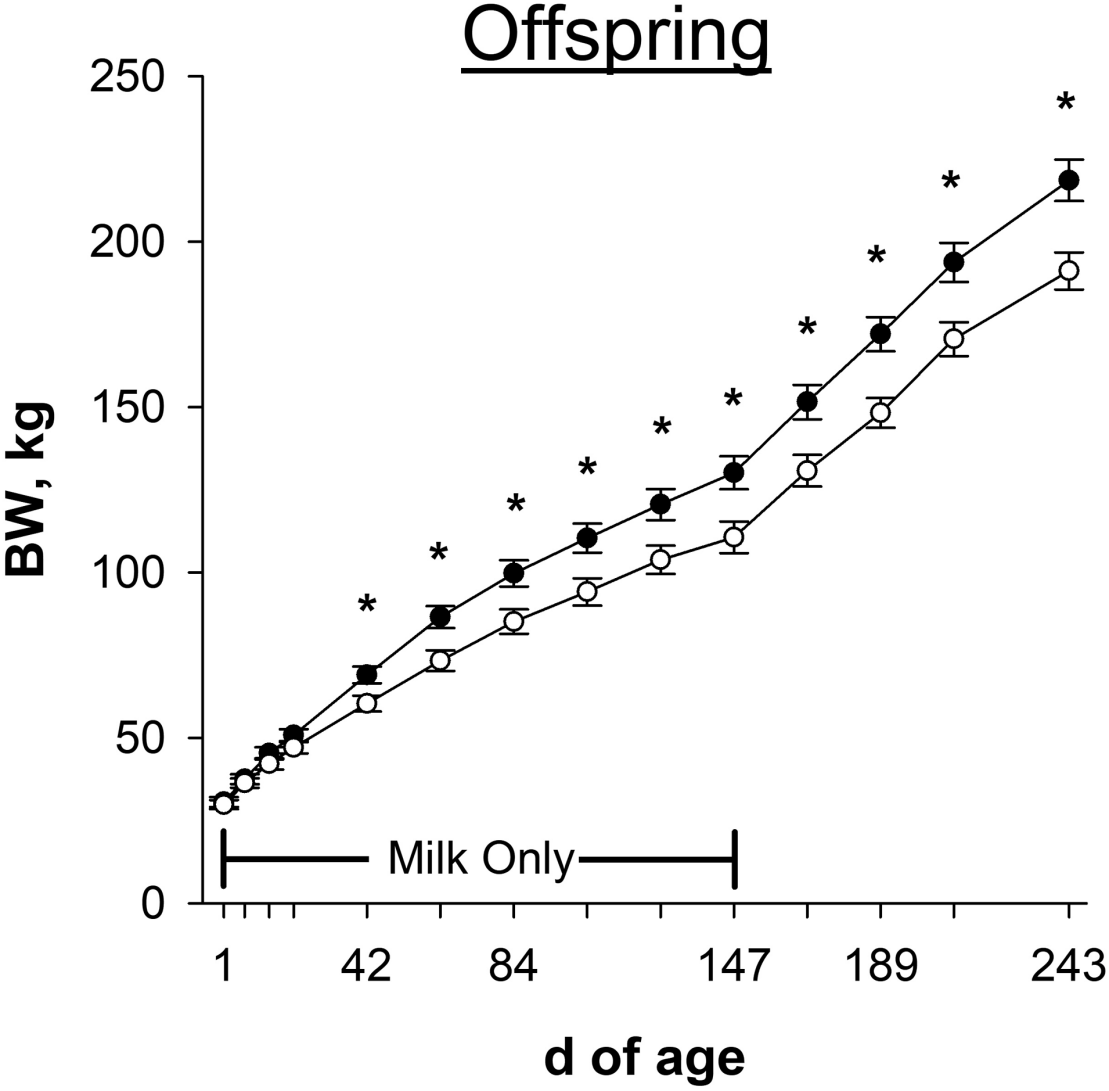
Effects of late gestational nutritional plane on calf body weight (BW) from birth until weaning. Solid circles (●) represent calves born to primiparous beef females individually-fed 100% (Control; *n* = 10 to 12) and open circles (○) represent calves born to primiparous beef females individually-fed 70% (Nutrient Restricted; *n* = 12 or 13) of estimated metabolizable energy and metabolizable protein requirements for maintenance, pregnancy, and growth from day 160 of gestation to parturition. All dams were individually-fed 100% of estimated metabolizable energy and metabolizable protein requirements for maintenance, lactation, and growth from parturition until day 149 of lactation and then managed in groups to meet estimated requirements until day 243 of lactation (weaning). Calves had access to milk only from days 1 to 149 of age, and then had access to milk and ad libitum tall fescue-based hay from days 150 to 243 of age. Least squares means ± SEM are presented. *Nutritional plane means differ (*P* ≤ 0.05).

Calf BW on days 7, 14, and 21 of age were not affected (*P* ≥ 0.14; Figure 4) by late gestational nutritional plane. By day 42 of age, calf BW diverged, where calves born to NR dams weighed 12.6% less (*P* = 0.02; 60.4 vs. 69.1 ± 2.5 kg) than calves born to CON. Calf BW remained different (*P* ≤ 0.02) through day 147 of age, at which point calves born to NR dams weighed 14.6% less (*P* = 0.01; 111 vs. 130 ± 5 kg) than CON. From days 1 to 147 of age, when calves consumed milk only, calves born to NR dams had lower (*P* = 0.02; Table 1) ADG than CON. This resulted from lower (*P* ≤ 0.02; data not shown) ADG from days 7 to 14 and days 21 to 63 of age in calves born to NR dams compared with CON. Calves born to NR dams weighed less (*P* ≤ 0.008) than CON at all timepoints while dams were group-fed and calves had access to ad libitum hay. At weaning (day 243 of age), calves born to NR dams weighed 12.8% less (*P* = 0.004; 191 vs. 219 ± 6 kg) than CON. From days 148 to 243 of age, when calves also consumed hay with their dams, calves born to NR dams tended to have lower (*P* = 0.08; Table 1) ADG than CON. This resulted from lower (*P* ≤ 0.05; data not shown) ADG from days 168 to 189 and days 210 to 243 of age in calves born to NR dams compared with CON.

**Figure 5.**
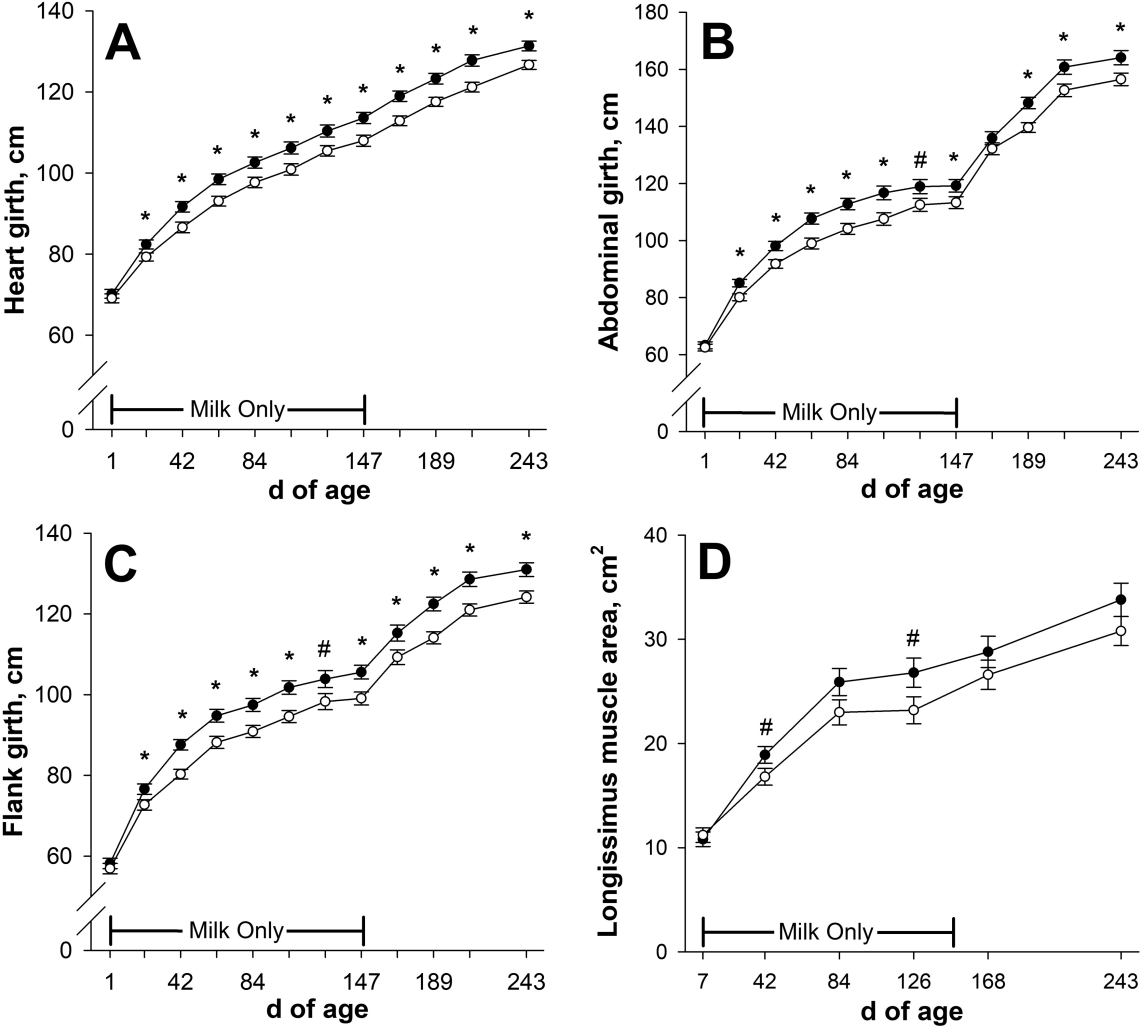
Effects of late gestational nutritional plane on calf heart girth (panel A), abdominal girth (panel B), flank girth (panel C), and longissimus muscle area (panel D) from birth until weaning. Solid circles (●) represent calves born to primiparous beef females individually-fed 100% (Control; *n* = 10 to 12) and open circles (○) represent calves born to primiparous beef females individually-fed 70% (Nutrient Restricted; *n* = 12 or 13) of estimated metabolizable energy and metabolizable protein requirements for maintenance, pregnancy, and growth from day 160 of gestation to parturition. All dams were individually-fed 100% of estimated metabolizable energy and metabolizable protein requirements for maintenance, lactation, and growth from parturition until day 149 of lactation and then managed in groups to meet estimated requirements until day 243 of lactation (weaning). Calves had access to milk only from days 1 to 149 of age, and then had access to milk and ad libitum tall fescue-based hay from days 150 to 243 of age. Least squares means ± SEM are presented. *Nutritional plane means differ (*P* ≤ 0.05). #Nutritional plane means tend to differ (0.05 < *P* ≤ 0.10).

Heart girth was less (*P* ≤ 0.05; Figure 5A) from days 21 to 243 of age in calves born to NR dams compared with calves born to CON. Calves born to NR dams had smaller (*P* ≤ 0.05; Figure 5B) abdominal girth from days 21 to 105, on day 147, and from days 189 to 243, and they tended to have smaller (*P* = 0.07) abdominal girth on day 126 of age compared with CON. Calves born to NR dams had smaller (*P* ≤ 0.04; Figure 5C) flank girth from days 21 to 105 and days 147 to 243 and tended to have smaller (*P* = 0.07) flank girth on day 126 of age compared with CON. Calf longissimus muscle area tended to be smaller (*P* ≤ 0.07; Figure 5D) in calves born to NR dams than CON on days 42 and 126 but was not affected (*P* ≥ 0.11) by late gestational nutritional plane on day 84, 168, or 243 of age.

**Figure 6.**
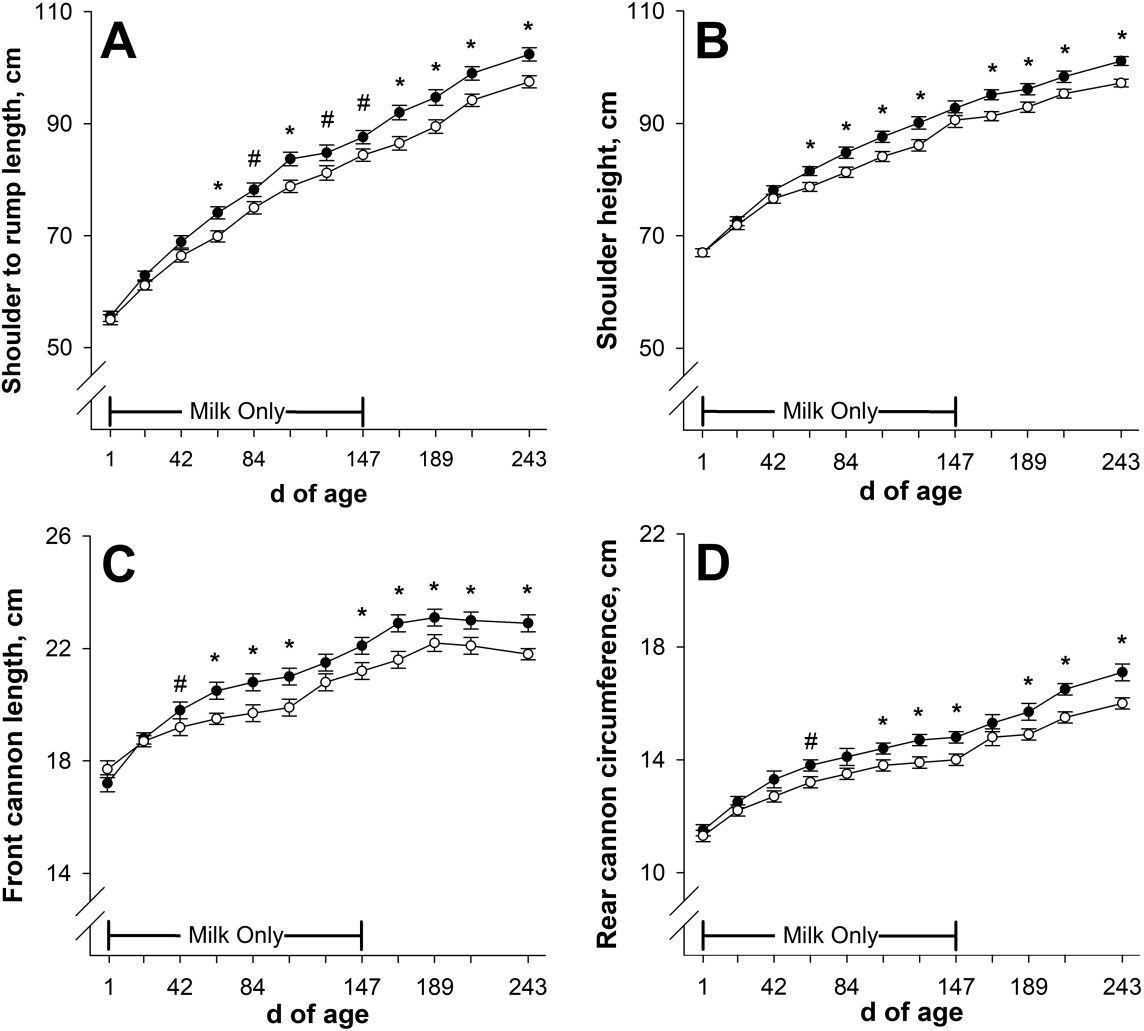
Effects of late gestational nutritional plane on calf shoulder to rump length (panel A), shoulder height (panel B), front cannon length (panel C), and rear cannon circumference (panel D) from birth until weaning. Solid circles (●) represent calves born to primiparous beef females individually-fed 100% (Control; *n* = 10 to 12) and open circles (○) represent calves born to primiparous beef females individually-fed 70% (Nutrient Restricted; *n* = 12 or 13) of estimated metabolizable energy and metabolizable protein requirements for maintenance, pregnancy, and growth from day 160 of gestation to parturition. All dams were individually-fed 100% of estimated metabolizable energy and metabolizable protein requirements for maintenance, lactation, and growth from parturition until day 149 of lactation and then managed in groups to meet estimated requirements until day 243 of lactation (weaning). Calves had access to milk only from days 1 to 149 of age, and then had access to milk and ad libitum tall fescue-based hay from days 150 to 243 of age. Least squares means ± SEM are presented. *Nutritional plane means differ (*P* ≤ 0.05). #Nutritional plane means tend to differ (0.05 < *P* ≤ 0.10).

Calf shoulder to rump length on days 21 and 42 of age were not affected (*P* ≥ 0.12; Figure 6A) by late gestational nutritional plane. On days 63, 105, and 168 to 243 of age, shoulder to rump length was less (*P* ≤ 0.01) in calves born to NR dams compared with calves born to CON. Shoulder to rump length tended to be less (*P* ≤ 0.08) on days 84, 126, and 147 of age in calves born to NR dams compared with CON. Calf shoulder height was not affected (*P* ≥ 0.21; Figure 6B) by late gestational nutritional plane on day 21, 42, or 147 of age. At all other timepoints from birth through weaning, shoulder height was less (*P* ≤ 0.03) in calves born to NR dams compared with CON. Calf front cannon length was not affected (*P* ≥ 0.14; Figure 6C) by late gestational nutritional plane on day 21 or 126 of age. Calves born to NR dams tended to have shorter (*P* = 0.09) front cannon length on day 42 and had shorter (*P* ≤ 0.05) front cannon length from days 63 to 105 and days 147 to 243 of age compared with CON. Calf rear cannon circumference was not affected (*P* ≥ 0.12; Figure 6D) by late gestational nutritional plane on day 21, 42, 84, or 168 of age. Calves born to NR dams tended to have smaller (*P* = 0.06) rear cannon circumference on day 63 and had smaller (*P* ≤ 0.04) rear cannon circumference from days 105 to 147 and days 189 to 243 of age compared with CON.

**Figure 7.**
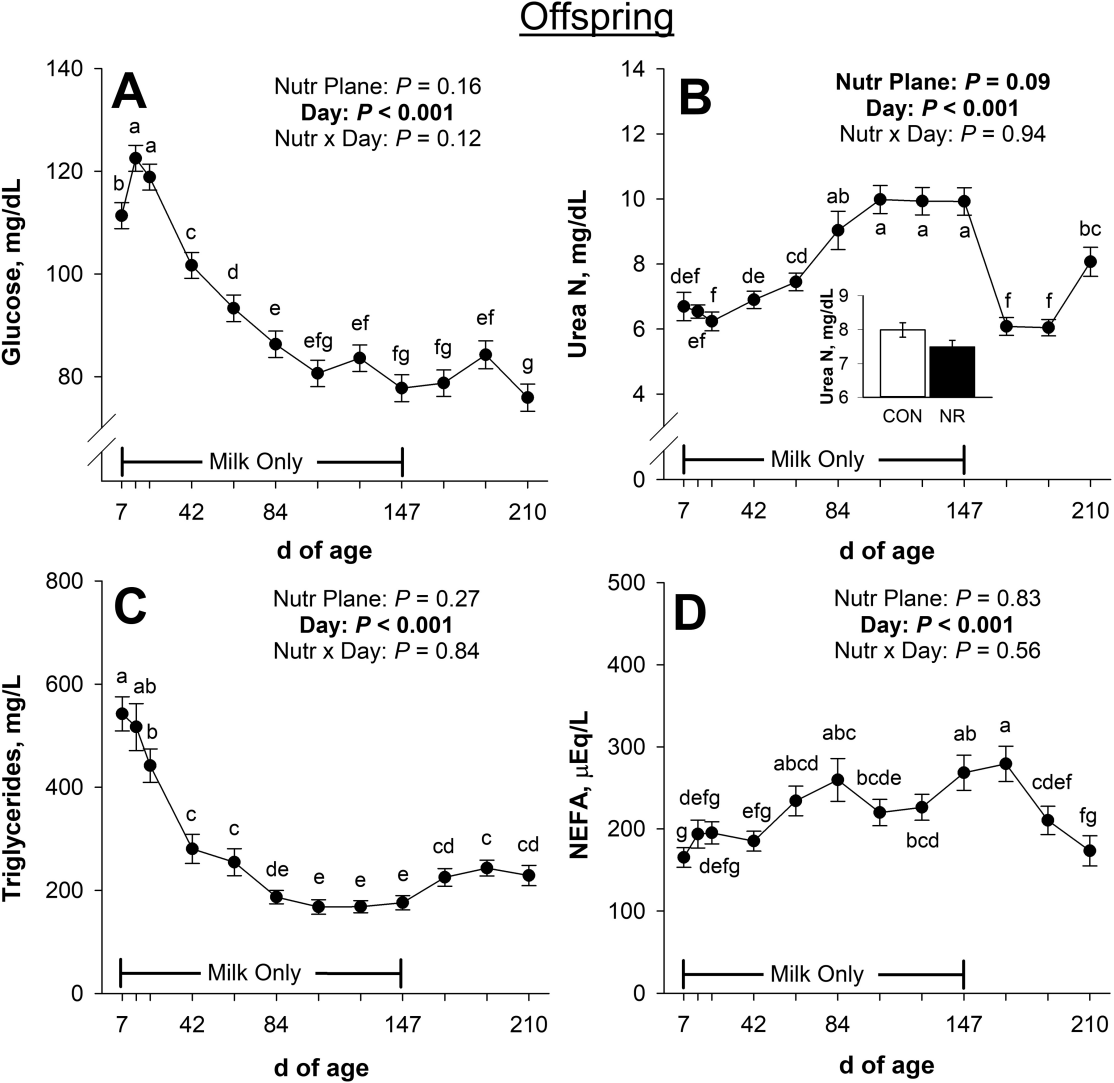
Effects of late gestational nutritional plane on pre-weaning calf plasma glucose (panel A), serum urea N (panel B), plasma triglycerides (panel C), and serum non-esterified fatty acids (NEFA; panel D) from days 7 to 210 of age. Solid circles (●) represent calves born to primiparous beef females individually-fed 100% (Control; *n* = 10 to 12) and open circles (○) represent calves born to primiparous beef females individually-fed 70% (Nutrient Restricted; *n* = 12 or 13) of estimated metabolizable energy and metabolizable protein requirements for maintenance, pregnancy, and growth from day 160 of gestation to parturition. All dams were individually-fed 100% of estimated metabolizable energy and metabolizable protein requirements for maintenance, lactation, and growth from parturition until day 149 of lactation and then managed in groups to meet estimated requirements until day 243 of lactation (weaning). Calves had access to milk only from days 1 to 149 of age, and then had access to milk and ad libitum tall fescue-based hay from days 150 to 243 of age. Least squares means ± SEM are presented. ^a,b,c,d,e,f,g^Means differ (*P* ≤ 0.05) for main effect of day.

Calf pre-weaning plasma glucose concentrations were not affected (*P* ≥ 0.12; Figure 7A) by the late gestational nutritional plane × day interaction or the main effect of nutritional plane, but there was an effect (*P* < 0.001) of day of age on plasma glucose. Plasma glucose increased (*P* < 0.001) from days 7 to 14, decreased (*P* ≤ 0.04) from days 21 to 84, and tended to decrease (*P* = 0.09) from days 84 to 105 of age. Additionally, calf plasma glucose tended to decrease (*P* = 0.08) from days 126 to 147 and decreased (*P* = 0.02) from days 189 to 210 of age.

The late gestational nutritional plane × day interaction did not affect (*P* = 0.94; Figure 7B) calf pre-weaning serum urea N concentrations. Pre-weaning, calves born to NR dams tended to have lower (main effect; *P* = 0.09) serum urea N than calves born to CON. Day of age also affected (*P* < 0.001) serum urea N. Serum urea N increased (*P* = 0.04) from days 21 to 42, tended to increase (*P* = 0.08) from days 42 to 63, and increased (*P* = 0.005) from days 63 to 84 of age. In addition, calf serum urea N decreased (*P* < 0.001) from days 147 to 168 but increased (*P* < 0.001) from days 189 to 210 of age.

Calf pre-weaning plasma triglyceride concentrations were not affected (*P* ≥ 0.27; Figure 7C) by the late gestational nutritional plane × day interaction or the main effect of nutritional plane. There was an effect (*P* < 0.001) of day of age on plasma triglycerides, where concentration decreased (*P* ≤ 0.01) from days 21 to 42 and days 63 to 84 but increased (*P* = 0.02) from days 147 to 168 of age.

Serum NEFA concentrations of pre-weaning calves were not affected (*P* ≥ 0.56; Figure 7D) by the late gestational nutritional plane × day interaction or the main effect of nutritional plane. Serum NEFA were affected (*P* < 0.001) by day of age. Serum NEFA increased (*P* = 0.01) from days 42 to 63 and tended to increase (*P* = 0.08) from days 126 to 147 of age. Additionally, serum NEFA decreased (*P* = 0.005) from days 168 to 189 and tended to decrease (*P* = 0.10) from days 189 to 210 of age.

## Discussion

### Maternal lactational performance and metabolism during individual feeding

We reported that during late gestation, control dams gained maternal BW, maintained BCS and backfat thickness, and were metabolically stable (Redifer et al., 2023). Conversely, nutrient restricted dams decreased maternal BW, BCS, and backfat thickness, had elevated NEFA concentrations, and had reduced circulating glucose, urea N, and triglycerides during late gestation. As a result, nutrient restricted dams were 63.6 kg BW and 2.0 BCS less than controls post-calving. Nutrient restricted dams prioritized nutrient delivery to fetal growth over maternal growth; thus, calf birth weight was not affected (Redifer et al., 2023).

Within normal production scenarios, it is not unusual for beef females to be nutrient restricted during late gestation but have improved nutrition post-calving, as cow–calf producers often align their calving season to optimize forage quantity and quality for the nutrient needs of the lactating dam. In the current study, dams from both late gestational nutritional planes were fed to meet their estimated nutrient requirements for maintenance, growth, and lactation. Control dams achieved the maternal growth that was projected based on the ME and MP allocated for gain, while nutrient restricted dams out-performed that projection. Primiparous beef females are expected to continue growing during their first lactation; however, the compensatory gain of previously nutrient restricted dams was not just skeletal growth and lean tissue accretion. Previously nutrient restricted dams gained body condition during this period, replenishing basal adipose reserves, while controls decreased body condition and backfat. Milk yield during the first 147 d of lactation was 15% less for nutrient restricted dams (Redifer et al., 2024), requiring less nutrients for milk production and leaving more nutrients available for maternal tissue gain.

Past late gestational nutrient restriction studies have consistently reported that heifers and cows underfed during pregnancy experienced greater postpartum gain when all dams were fed adequately after calving (Jordan et al., 1968; Bellows et al., 1982; Anthony et al., 1986; Wiley et al., 1991). Individual intake data was not available in those studies to determine if this was due to greater intakes or more efficient utilization of nutrients, but our data suggest that it was likely increased metabolic efficiency. Even when adjusting their BW for maintenance requirements to a BCS 5.0, previously nutrient restricted dams consumed similar or less ME and CP during the first 147 d of lactation. Average ME and CP intakes of previously nutrient restricted dams were within 3.7% of controls during individual feeding; thus, lactational intake differences were minimal from a production standpoint. In agreement, ewes nutrient restricted during gestation experienced greater ADG, even when consuming less DMI, during the first 20 d of lactation (Meyer et al., 2010). This was also attributed to improved efficiency and a reduction of nutrients being partitioned to lactation due to decreased milk yield (Meyer et al., 2011).

Growing animals that were previously underfed stabilizing at a lower basal metabolic rate and experiencing compensatory growth when provided with plentiful nutrients is well known (Carstens et al., 1991; Drouillard et al., 1991). Although growing steers partitioned nutrients to protein accretion rather than fat deposition during compensatory growth (Carstens et al., 1991), our lactating first-parity dams gained 0.68 BCS when refed to meet lactational requirements. Efficiency of energy and protein retention after prolonged nutrient restriction has been demonstrated in both non-pregnant, non-lactating (Freetly and Nienaber, 1998) and pregnant (Freetly et al., 2008) beef cows.

It is likely that the postpartum BW gain of all dams was partially reflective of increases in organ mass due to lactation, and the compensatory growth of previously nutrient restricted dams included greater increases in organ masses. Changes in gastrointestinal organs and liver masses were observed within 21 d of altering nutritional plane (Burrin et al., 1990). The physiological switch from gestation to lactation and the inherent increase in voluntary DMI also increase visceral organ masses (Reynolds et al., 2004). Nutrient restricted ewes had smaller absolute gastrointestinal tract and liver masses (that were similar relative to BW) at parturition than adequately-fed ewes, but after adequate feeding for 20 d of lactation, previously nutrient restricted ewes had greater visceral organ masses relative to BW (Meyer et al., 2012b).

We reported that the act of parturition resulted in elevated glucose and decreased triglycerides (although both were lower for nutrient restricted dams) at 1-h post-calving, and control NEFA spiked to a similar concentration as observed for nutrient restricted dams during late pregnancy (Redifer et al., 2023). In the current dataset, the day 1 of lactation sampling time occurred between 6 and 30 h postpartum, but some of the metabolic effects of parturition still existed, as glucose and NEFA were still stabilizing.

Previously nutrient restricted dams responded quickly to being fed to meet estimated nutrient requirements, with circulating triglycerides and NEFA returning to similar concentrations as control dams by day 42 of lactation. Circulating NEFA are sensitive to refeeding and have returned to pre-fasting concentrations within 2 d of ending long-term underfeeding (Chilliard et al., 1998), likely due to the priority to replenish adipose stores and suppress all but basal lipolysis upon refeeding (Vernon, 1992). Interestingly, nutrient restricted dams in the current study had lower NEFA concentration on day 21 of lactation than controls. In agreement with our findings, beef cows that calved at a BCS 4 had lower circulating NEFA and greater lipogenic enzyme activity during the first 2 mo of lactation than cows calving at a BCS 6 (Lake et al., 2006a, 2006b). The BCS 4 cows in Lake et al. (2005) maintained body condition during early lactation, and milk production was not affected by BCS at calving. In the current study, control dams had greater milk production (Redifer et al., 2024), which most likely made them more sensitive to the lipolytic demands and adipose mobilization of early lactation (Bauman and Currie, 1980).

For the remainder of individual feeding, circulating glucose, triglycerides, and NEFA were relatively stable and unaffected by late gestational nutritional planes. Beef cattle data demonstrating how quickly maternal metabolism recovers after gestational undernutrition when refed during lactation are sparse, but previous research is in agreement (Valiente et al., 2018). We reported that control female NEFA concentrations during late gestation were approximately 200 µEq/L (Redifer et al., 2023), which decreased and stabilized at approximately 100 µEq/L during early lactation for all dams. This suggests that the strain on adipose mobilization was greater due to nutrient demands of late gestational fetal growth than milk synthesis in early lactation, in agreement with Sletmoen-Olson et al. (2000) but in stark contrast to high-producing dairy cows (Bauman and Currie, 1980). The multiple increases in serum urea N concentrations during the first 147 d of lactation were not expected given that protein intake was controlled during that period. Low circulating NEFA concentrations suggest protein was not being deaminated to meet energetic needs, but perhaps protein needs were overestimated relative to lactational demands, and excess amino acids were deaminated.

### Maternal lactational performance and metabolism during group feeding

After day 149 of lactation, dams and calves were fed in large drylots, which resulted in all dams initially losing BW, as well as increased variation in BW and circulating metabolites. This was likely caused by less consistent feed intake behaviors relative to BW and blood sampling time, social hierarchy changes due to group feeding, supplementation to the group average nutrient requirements, and greater exposure to environmental stressors. For many of the dams, days 150 to 243 of lactation represented late January through late April, and they experienced multiple prolonged periods below their lower critical temperature. Thus, there may have been a reliance on tissue reserves (elevated NEFA concentrations) and amino acid deamination to meet the greater associated energy demands, resulting in weight loss (Wagner, 1988).

We hypothesized that previously nutrient restricted dams fed to meet estimated nutrient requirements during lactation would have similar BW and body condition to controls by weaning. All dams decreased BW during group feeding; however, control dams had greater BW and BCS losses. Despite this, previously nutrient restricted dams still had lower BCS, less backfat thickness, and less BW (numerically) at weaning. The control dam removed after day 168 of lactation due to calf mortality was approximately 70 kg heavier than the group average; therefore, her removal and the increased variation described above exaggerated the convergence in dam BW at weaning.

In past studies where late gestational nutrient restriction resulted in post-calving maternal performance differences less severe than we observed, dam BW converged by breeding (Anthony et al., 1986) or weaning (Bellows et al., 1982). Weight cycling and deferring feed resources during gestation and lactation resulted in maternal BW and BCS differences nearly as severe as we reported, but both primiparous (Freetly et al., 2005) and multiparous (Freetly et al., 2000) dams recovered to a similar or greater BW by subsequent breeding. Other gestational nutrient restriction experiments more severe in degree or length reported that maternal performance did not recover by weaning (Jordan et al., 1968; Cafe et al., 2006). Nutritional plane can be oscillated during the annual production cycle and the dam may recover; however, there appears to be a threshold where the insult to maternal BW and body composition may be long-term. The concept that altering nutritional plane during critical physiological stages, especially in growing first-parity females, may impact stayability within the herd and lifetime cow productivity deserves continued investigation.

### Dam reproductive performance

The current study was not designed with the statistical power to detect differences in reproductive performance, but late gestational nutritional plane did not appear to impact conception rates. When beef cows calved at a thin BCS but were in a positive energy balance postpartum, they had improved estrus response (Richards et al., 1986) and pregnancy rates (Houghton et al., 1990) compared with poor lactational nutrition. The successful overall conception rates observed in the current study were largely attributed to individual feeding of all dams to meet estimated nutrient requirements during lactation. In a production setting, improved postpartum nutrition may not occur during droughts or times of poor forage nutrient availability. Moreover, it is unlikely that lactational nutrition would improve so drastically immediately after calving; thus, poorer overall reproductive performance of previously nutrient restricted beef females could be expected.

### Calf pre-weaning growth and metabolism consuming milk only

We reported that calf BW and size at birth were not affected by late gestational nutrient restriction (Redifer et al., 2023), but fetal growth is not necessarily indicative of fetal development. Late gestational nutrient restriction resulted in less vigorous calves with more indicators of stress and less red blood cells over the first 48 h of life (Wichman et al., 2023). Calves born to nutrient restricted dams had greater serum immunoglobulin concentrations at 48 h of age, but calves born to dams on both late gestational nutritional planes achieved successful transfer of passive immunity (Wichman et al., 2023).

For the first 5 mo of life, calves had access to milk only to prevent them from compensating for decreased milk nutrient availability through consumption of feed or forage as often occurs in more extensive studies. Calves born to nutrient restricted dams had lower ADG from birth until day 147 of age, resulting in calf BW diverging by day 42 of age and a 15% deficit in BW at day 147 of age. In gestational nutrient restriction models where the calves are reared naturally by their dams, it is difficult to delineate the prenatal and postnatal effects on pre-weaning growth. Given the similar reduction (15% less) in milk yield during this same period for nutrient restricted dams, which resulted in 29% less total protein, 21% less total triglycerides, 14% less total lactose, and 19% less total urea N available for their calves to consume (Redifer et al., 2024), milk yield may have driven this divergence in growth. There is a strong correlation between milk yield and weaning weight (Reynolds et al., 1978), explaining greater than 50% of the variation in pre-weaning ADG in 1 study (Arthur et al., 1997). In the current study, when separated by late gestational nutritional plane, average milk yield and calf ADG during the first 5 mo of age were not correlated (*r* = 0.13; *P* = 0.70) for control calves but were strongly correlated (*r* = 0.69; *P* = 0.01) for nutrient restricted calves.

Still, the idea that developmental programming effects on fetal development contribute to poor postnatal outcomes (reviewed by Meyer et al., 2012a and Robinson et al., 2013) cannot be ruled out in the current study. After mid- and late gestational undernutrition, lambs were separated from their dams and raised on ad libitum milk replacer while their dams were mechanically milked. Both slower lamb growth rates (Meyer et al., 2010) and reduced milk yield (Meyer et al., 2011) were observed independently, supporting the hypothesis that gestational nutrient restriction has two-fold effects on both prenatal (programming metabolic capacity and growth) and postnatal (affecting milk yield and nutrient composition) nutrient delivery.

To our knowledge, there is no past research investigating the effect of gestational nutrition on such a vast array of serially-collected beef calf size measures pre-weaning. Calf BW divergence was preceded by girth measures separating on day 21 of age. Heart girth is known to be a reliable predictor of BW in pre-weaning beef (Rotondo, 2021) and dairy (Wilson et al., 1997) calves. In calves fed milk only, growth of the reticulorumen and omasum were roughly proportional to the increase in calf BW (Warner et al., 1956). It is likely that the divergence in abdominal girth and flank girth measures is reflective of gastrointestinal organ growth relative to milk availability, which foreshadowed BW changes.

Skeletal size measures predominantly diverged after calf BW at day 63 of age. When partitioning nutrients, bone is prioritized over soft carcass tissues (Hammond, 1944), so it is believed that bone growth is more resistant to the negative impacts of undernutrition in immature animals. Lower milk replacer feeding rate in dairy calves slowed skeletal growth in Orellana Rivas et al. (2020) and Shiasi Sardoabi et al. (2021), but did not affect body length or shoulder height in Bartlett et al. (2006).

Calf longissimus muscle area was quite variable and thus only tended to be smaller in calves born to nutrient restricted dams on days 42 and 126 of age. Late gestational undernutrition resulted in smaller longissimus muscle area at weaning in Cafe et al. (2006) but not in Long et al. (2021). Cafe et al. (2006) reported that longissimus muscle area relative to calf BW at weaning was greater in calves of undernourished dams, suggesting greater priority compared with overall BW gains, but this was not observed in the current study (data not shown).

We reported that neonatal circulating glucose, urea N, triglycerides, and NEFA during the first 2 d of age were not affected by late gestational nutritional plane (Wichman et al., 2023). Despite the large discrepancy in milk nutrients available (Redifer et al., 2024), calf metabolic status pre-weaning was also minimally affected by late gestational nutritional plane. Similarly, feeding dairy calves greater amounts of milk replacer did not alter circulating glucose, urea N, triglycerides, or NEFA (Orellana Rivas et al., 2020). Despite this, greater milk replacer feeding rates increased circulating glucose and triglycerides and decreased urea N in dairy calves (Bartlett et al., 2006; Schäff et al., 2016). Circulating glucose concentrations were lower (*P* ≤ 0.03) on days 14 and 63 of age for calves born to nutrient restricted dams compared with controls (if interaction F-test was unprotected), likely due to decreased milk lactose available. Late gestational undernutrition also resulted in reduced milk production and less circulating glucose in pre-weaning beef calves in the study by Ryley and Gartner (1962).

Pre-ruminant neonatal calves in this study exhibited the innate desire to consume solid feeds, as they often ate sawdust bedding and began ruminating by 30 to 40 d of age even without ready access to feed or forage. Calves consuming solid feed shift towards a functional rumen within the first few weeks of life, but this is delayed when consuming milk only (Lengemann and Allen, 1959). Calf circulating glucose and triglyceride concentrations decreased from days 14 to 84 of age, at which point they remained fairly stable at concentrations appropriate for adult ruminants through weaning. High neonatal calf glucose concentrations that decline early in life were described by Lambert et al. (1955), irrespective of solid feed provision. Calf serum urea N concentrations increased during the first 3 mo of age and then plateaued until the end of the milk-only period. Not surprisingly, this pattern of calf serum urea N strongly resembled milk urea N (Redifer et al., 2024) and dam serum urea N concentrations during this time. Calf NEFA concentrations were erratic and without pattern from days 7 to 147 of age. This is likely a result of the young, growing animals being constrained to milk only, with limited available nutrients, but without adequate adipose reserves to mobilize.

### Calf pre-weaning growth and metabolism with access to hay

Once dams and calves were moved into drylots and calves were allowed to consume ad libitum hay, calves raised by dams on both late gestational nutritional planes experienced greater ADG. This suggests that consuming milk only during the first 5 mo of age did not provide the nutrients needed for maximal growth in the pre-weaning beef calf. Along with greater ADG, calf abdominal girth and flank girth increased at a faster rate in the drylots, indicative of increased gut fill and further ruminal development with access to forage. The greater nutrient availability with access to hay resulted in decreased circulating NEFA and urea N. This was likely due to decreased dependence on body stores and greater fermentable carbohydrates available for the incorporation of urea N into microbial protein synthesis. Calves born to nutrient restricted dams did not compensate for lower milk production with additional hay consumption, as they still weighed 13% less than controls at weaning.

Similar to our results, late gestational nutrient restriction did not affect calf birth weight, but calf performance diverged by weaning (Hodge et al., 1976; Stalker et al., 2006); however, milk production was not measured in those studies. Late gestational undernutrition also decreased calf birth weight and pre-weaning growth, accompanied by reduced (Ryley and Gartner, 1962; Corah et al., 1975; Kroker and Cummins, 1979) or similar (Corah et al., 1975) milk production. Wiley et al. (1991) reported no differences in birth weight, milk production, or weaning weight. In other instances, nutrient restriction resulted in birth weight but not weaning weight differences (Bellows and Short, 1978; Spitzer et al., 1995; Long et al., 2021), suggesting either gestational nutrition did not negatively impact milk production or additional forage consumption allowed for compensation in BW. There are many possible factors including parity, breed, calf sex, calving season, and environmental conditions that likely affected prenatal and postnatal nutrient delivery in these studies, resulting in inconsistent calf pre-weaning results. Decreased pre-weaning growth, as a result of gestational nutrient restriction, has contributed to later-life effects on feedlot performance and carcass composition (Greenwood and Cafe, 2007; Larson et al., 2009) and daughter puberty status and fertility (Martin et al., 2007; Funston et al., 2010).

### Pre-weaning calf health

Beyond the calf mortality due to congenital renal dysplasia and complications from surgical castration (both controls and unrelated to nutritional plane), there was minimal calf morbidity observed from birth until weaning. One calf (control) was administered electrolytes for scours, 2 calves (1 nutrient restricted and 1 control) were treated with antibiotics for navel infections, and 2 calves (1 nutrient restricted and 1 control) were given antibiotics for fever determined by rectal temperature greater than 40.5°C. Gestational undernutrition research in more extensive settings observed greater death loss in the first 2 wk of life (Kroker and Cummins, 1979) and an approximately 20 to 30% reduction in calves alive at weaning (Hight, 1966; Corah et al., 1975), largely attributed to starvation and scours. It is plausible that if our experiment was not conducted in such a controlled manner with close monitoring of calf health, or if it had occurred in cold, wet conditions (spring-calving rather than fall-calving), then a disparity in calf morbidity could have existed and even further exacerbated our findings on calf pre-weaning growth and metabolism.

Additionally, consuming milk only induced acute hypomagnesemic tetany in 1 calf (nutrient restricted, likely unrelated to nutritional plane), which resulted in the determination that most calves raised by dams on both late gestational nutritional planes were at least marginally magnesium deficient (low serum Mg concentrations). Duncan et al. (1935) first reported that calves fed milk only for extended periods of time develop hypomagnesemia, due to both a dietary deficiency of magnesium in milk and properties specific to the milk diet that decrease magnesium retention efficiency (Smith, 1957). This was unexpected due to the rare occurrence of calves consuming milk only for as long as 5 mo without access to forage (beef calves) or provision of starter (dairy calves) in current production systems. After the case of tetany, drenching with Mg sulfate during individual feeding and access to hay and mineral supplementation in the drylots alleviated this issue, and no additional calves exhibited clinical symptoms of hypomagnesemic tetany.

## Conclusions

First-parity beef females that were nutrient restricted during late gestation and then individually-fed to meet their estimated nutrient requirements during lactation recovered quickly metabolically, experienced compensatory growth, and replenished basal adipose reserves, yet contrary to our hypothesis, still had less body condition at weaning than controls. In a production scenario, limited nutrient availability may prevent adequate nutrition during lactation, which would likely result in altered maternal BW and body composition long-term, poor reproductive performance, and ultimately decreased longevity. Although calf birth weight was not affected, calf pre-weaning BW, girth measures, and skeletal size all diverged by day 63 of age while consuming milk only. Calves born to nutrient restricted dams weighed less at weaning, likely due in large part to decreased milk production (Redifer et al., 2024), but calf metabolic status was only minimally altered pre-weaning. In more extensive settings, incidence of morbidity and mortality would likely increase, further diminishing the kilograms of calf weaned per female exposed. In summary, first-parity beef females that were nutrient restricted during late gestation prioritized partitioning nutrients to maternal growth and energy reserves over milk production during lactation, yet dams were thinner at weaning, and pre-weaning calf growth was slowed.

## Abbreviations

ADF: acid detergent fiber
ADG: average daily gain
AI: artificial insemination
BCS: body condition score
BF: backfat
BW: body weight
CP: crude protein
DDGS: dried distillers' grains with solubles
DM: dry matter
DMI: dry matter intake
ME: metabolizable energy
MP: metabolizable protein
NDF: neutral detergent fiber
NEFA: non-esterified fatty acids
